# Breeding a new *Ganoderma lucidum* strain with increased contents of individual ganoderic acids by mono–mono crossing of genetically modified monokaryons

**DOI:** 10.3389/fmicb.2024.1410368

**Published:** 2024-05-30

**Authors:** Ding-Xi Zhou, Xiang-Ming Kong, Xiong-Min Huang, Na Li, Na Feng, Jun-Wei Xu

**Affiliations:** ^1^Faculty of Life Science and Technology, Kunming University of Science and Technology, Kunming, China; ^2^School of Pharmacy, East China University of Science and Technology, Shanghai, China; ^3^Faculty of Science, Kunming University of Science and Technology, Kunming, China; ^4^Institute of Edible Fungi, Shanghai Academy of Agricultural Sciences, Key Laboratory of Edible Fungi Resources and Utilization (South), Shanghai, China

**Keywords:** breeding, *Ganoderma*, ganoderic acids, genetic engineering, mono–mono crossing

## Abstract

Ganoderic acids (GAs) are major functional components of *Ganoderma lucidum*. The study aimed to breed a new *G. lucidum* strain with increased contents of individual GAs. Two mating-compatible monokaryotic strains, G. 260125 and G. 260124, were successfully isolated from the dikaryotic *G. lucidum* CGMCC 5.0026 via protoplast formation and regeneration. The *Vitreoscilla* hemoglobin gene (*vgb*) and squalene synthase gene (*sqs*) were overexpressed in the monokaryotic G. 260124 and G. 260125 strain, respectively. Mating between the G. 260124 strain overexpressing *vgb* and the G. 260125 strain overexpressing sqs resulted in the formation of the new hybrid dikaryotic *G. lucidum* strain sqs-vgb. The maximum contents of ganoderic acid (GA)-T, GA-Me, and GA-P in the fruiting body of the mated sqs-vgb strain were 23.1, 15.3, and 39.8 μg/g dry weight (DW), respectively, 2.23-, 1.75-, and 2.69-fold greater than those in *G. lucidum* 5.0026. The squalene and lanosterol contents increased 2.35- and 1.75-fold, respectively, in the fruiting body of the mated sqs-vgb strain compared with those in the *G. lucidum* 5.0026. In addition, the maximum expression levels of the *sqs* and lanosterol synthase gene (*ls*) were increased 3.23- and 2.13-fold, respectively, in the mated sqs-vgb strain. In summary, we developed a new *G. lucidum* strain with higher contents of individual GAs in the fruiting body by integrating genetic engineering and mono–mono crossing.

## 1 Introduction

*Ganoderma lucidum* is a tetrapolar species as indicated by Adaskaveg and Gilbertson (1986). This means that the development of a dikaryotic mycelium after the fusion of two compatible monokaryons, as well as fruiting and sexual reproduction via the formation of basidiospores on the established dikaryon, are regulated by two distinct mating type loci, matA and matB. It has been used in Asia to improve health and treat various diseases for over 2000 years (Bishop et al., 2015; Hsu and Cheng, 2018). *G. lucidum* has diverse biological activities including immunomodulation, anticancer, antiaging, and anti-inflammatory effects (Xu et al., 2010b; Ahmad et al., 2022). Owing to its nutritional and medicinal properties, *G. lucidum* has received widespread attention from academic and industrial communities in recent years due to its nutritional and medicinal properties. Ganoderic acids (GAs) are lanostane-type triterpenes found in mycelia, spores, and fruiting bodies, and are among the major functional components of *G. lucidum*. GAs exhibit numerous pharmacological activities. For example, individual ganoderic acid (GA)-Me and GA-T inhibit the growth and metastasis of lung cancer cells (Tang et al., 2006; Wang et al., 2007), GA-S induces the apoptosis of HeLa cells (Liu and Zhong, 2011), and GA-P inhibits the release of TNF-a and IL-6 in RAW 264.7 macrophages (Feng et al., 2023).

The market demand for *G. lucidum* is increasing due to its therapeutical actions and health-promoting benefits. The annual output of the *Ganoderma* industry is estimated to be US $2.5 billion (Bishop et al., 2015; Hsu and Cheng, 2018). As the consumption of *Ganoderma* products largely depends on their health and medicinal benefits, the contents of the bioactive components in the fruiting body of *G. lucidum* are of considerable interest to researchers and consumers. However, the GA content in the fruiting body of *G. lucidum* is very low, and these mushrooms cannot meet the market demand (Ye et al., 2018). Therefore, it is important to develop new *G. lucidum* strains with increased GA contents in their fruiting bodies.

Various breeding methods have been used to improve *G. lucidum* strains. Protoplast fusion, mutagenesis, mycelial mating, and genetically engineered transformation are the currently available techniques (Qi et al., 2003; Xu and Zhong, 2015; Liu et al., 2017; Raman et al., 2021; Xu, 2021). Among these approaches, genetic engineering has been successfully applied to construct new strains with improved bioactive component contents in the mycelia of *G. lucidum* (Zhang et al., 2017b; Zhao et al., 2022). However, manipulating multiple genes remains challenging in *G. lucidum* because of the limited number of available selectable markers (Xu et al., 2023). Among the available breeding approaches, mycelial mating of compatible monokaryotic strains is the most widely used method and results in the creation of various new strains (Kim et al., 2013; Wang et al., 2018; Lin et al., 2021), including strains of *G. lucidum*, which has relatively high polysaccharide and triterpenes contents in its fruiting body (Liu et al., 2017). This technique involves mating two monokaryotic compatible strains, whose hyphae can fuse and give rise to a dikaryotic mycelium (Ramirez et al., 2000). Nevertheless, the benefits of hybridization depend on the specific parent strains involved in mono–mono crossing (Vecchio et al., 2012; Avin et al., 2016). Careful selection and evaluation of parental strains are essential for obtaining the desired traits in mated strains. Mono–mono crossing of genetically engineered parental strains places both modified genes in a dikaryotic strain with different recombinant genomes. Therefore, the combination of genetic engineering and mycelial mating may be a promising approach to breeding *G. lucidum*. However, this approach has not yet been attempted in *Ganoderma*.

Previous studies have shown that the heterologous expression of the *vgb* or the GA biosynthesis-related gene *sqs* effectively improves the contents of individual GAs in submerged cultures of *G. lucidum* (Zhou et al., 2014; Li et al., 2016b). The expression of VHb, an oxygen-binding heme protein, may enhance oxygen utilization and accelerate oxygen-requiring steps in the GA biosynthesis pathway in *G. lucidum*. The overexpression of the SQS gene results in an increased accumulation of individual GAs and up-regulation of the LS gene in *G. lucidum*. However, the effects of manipulating these genes on GA accumulation in the *G. lucidum* fruiting body have not been reported. Thus, it is interesting to investigate whether mycelial mating of the monokaryotic strain overexpressing *vgb* and the compatible strain overexpressing *sqs* can create a new hybrid strain with a high content of individual GAs in the fruiting body.

In the present study, we isolated two monokaryotic strains from a dikaryotic *G. lucidum* strain via protoplast formation and regeneration. The *vgb* and *sqs* were overexpressed in the two isolated monokaryotic strains of *G. lucidum*, respectively. A new hybrid dikaryotic strain was subsequently obtained by mono–mono crossing of two genetically engineered monokaryotic strains. Moreover, the contents of individual GAs, the accumulations of intermediates, and the transcription levels of three biosynthetic genes were investigated at three different developmental stages in the control and newly generated hybrid strains, confirming the development of a new *G. lucidum* hybrid strain with high contents of individual GAs in the fruiting body.

## 2 Materials and methods

### 2.1 Strains and culture conditions

The dikaryotic *G. lucidum* strain CGMCC 5.0026 was obtained from the China General Microbiological Culture Center. The *G. lucidum* mycelia were cultured on potato dextrose medium in the dark at 30°C. Preculturing of *G. lucidum* mycelia was performed as described by Xu et al. (2010a, 2012). The solid culture medium for *G. lucidum* fruiting body growth comprised the following components: 20% wheat bran, 58% cotton seed hull, 20% sawdust, 1% CaSO_4_, and 1% sucrose. The moisture content was adjusted to 60% by adding distilled water. The cultivation bags containing 500 g of solid culture medium were inoculated with 30 mL of preculture broth and placed in an incubator at 30°C until filled with mycelia. *G. lucidum* fruiting body growth of *G. lucidum* was promoted at 30°C with approximately 90% relative humidity and 800 lux light intensity. The *Escherichia coli* JM109 strain used for plasmid construction was cultured on Luria-Bertani agar plates at 37°C.

### 2.2 Preparation and regeneration of *Ganoderma lucidum* protoplasts

*Ganoderma lucidum* mycelia were collected by centrifugation and rinsed with sterilized water. The collected mycelia were suspended in 2.0% lywallzyme (Guang Dong Institute of Microbiology, China) supplemented with 0.6 M mannitol and 0.1 M sodium citrate (pH 5.6). After incubating for 2.5 h, the protoplasts were purified by filtration through absorbent cotton and washed with 0.6 M mannitol by centrifugation at 1,500 g for 5 min. The protoplasts were allowed to regenerate at 30°C in complete yeast medium (CYM) supplemented with 1% maltose, 2% glucose, 0.2% yeast extract, 0.2% tryptone, 0.05% MgSO_4_, 0.46% KH_2_PO_4_, 0.6 M mannitol, and 1.5% agar. Viable colonies originating from the regenerated protoplasts were picked and transferred to potato dextrose agar (PDA) slants.

### 2.3 Genetic transformation of *Ganoderma lucidum* and identification of transformants

Genetic transformation of *G. lucidum* protoplasts was performed as previously described (Yu et al., 2014). Following the transformation of the plasmids pJW-EXP-SQS (Zhou et al., 2014) and pJW-EXP-In-Op-vgb (Wang et al., 2021) into the monokaryotic G. 260125 and G. 260124 strains, respectively, the G. 260125-sqs and G. 260124-vgb transformants were screened on CYM plates containing 2 mg/L carboxin. The obtained carboxin-resistant transformants were verified by genome PCR amplification of the fusion fragment containing the glyceraldehyde-3-phosphate dehydrogenase gene (*gpd*) promoter and the target gene (*sqs* or *vgb*). The following primers were used for genome PCR amplification: gpd-F, 5′-TCCAAAGCCGCTCTCATGGC-3′ and sqs-R, 5′-AGTTGCCTGTCCTTTTCTTT-3′; gpd-F, 5′-TCCAAAGCCGCTCTCATGGC-3′ and vgb-R, 5′-CCCGGGTCACTCGACGGCCTGGG-3′. *Vitreoscilla* hemoglobin activity was determined by CO-difference spectrum analysis as previously described (Li et al., 2016a; Wang et al., 2021).

### 2.4 Mono–mono crossing between different monokaryotic *Ganoderma lucidum* strains

Mycelia of the two different monokaryotic strains were inoculated 10 mm apart on PDA plates and incubated at 30°C until they merged. The mycelia from the contact area between the two monokaryotic mycelia were subcultured onto fresh PDA plates in the dark. Protoplasts from the merged mycelia were regenerated on CYM plates. Dikaryotic colonies from regenerated protoplasts were screened by observation of clamp connections and SNP-PCR analysis (Sun et al., 2021). The primers SNP-F (5′-ATGACACGGACCTCATAGCCT-3′) and SNP-R (5′-ATGTCGCTCAGCCTGTCCAA-3′) were used for genome PCR.

### 2.5 Transcriptional analysis of GA biosynthetic genes using quantitative real time-PCR

Total RNA was extracted from *G. lucidum* with TRI zol reagent (Invitrogen, Carlsbad, CA) and reverse-transcribed to first-strand cDNA using PrimeScript RT reagent Kit (Takara, Dalian, China) following the manufacturer’s instructions. The relative transcription levels of the 3-hydroxy-3-methyglutaryl coenzyme A reductase gene (*hmgr*), *sqs*, and the lanosterol synthase gene (*ls*) in the mated sqs-vgb strain were determined via quantitative real time-PCR (qRT-PCR) analysis previously described (Zhang et al., 2017a). Gene transcription from the CGMCC 5.0026 strain was defined as 1.0, and the transcription levels of genes from the sqs-vgb strain were expressed as fold changes compared with those in the control strain. Relative expression levels were calculated using the 2^ΔΔCt^ method.

### 2.6 Determination of the contents of individual GAs and accumulations of squalene and lanosterol

**Figure 1:**
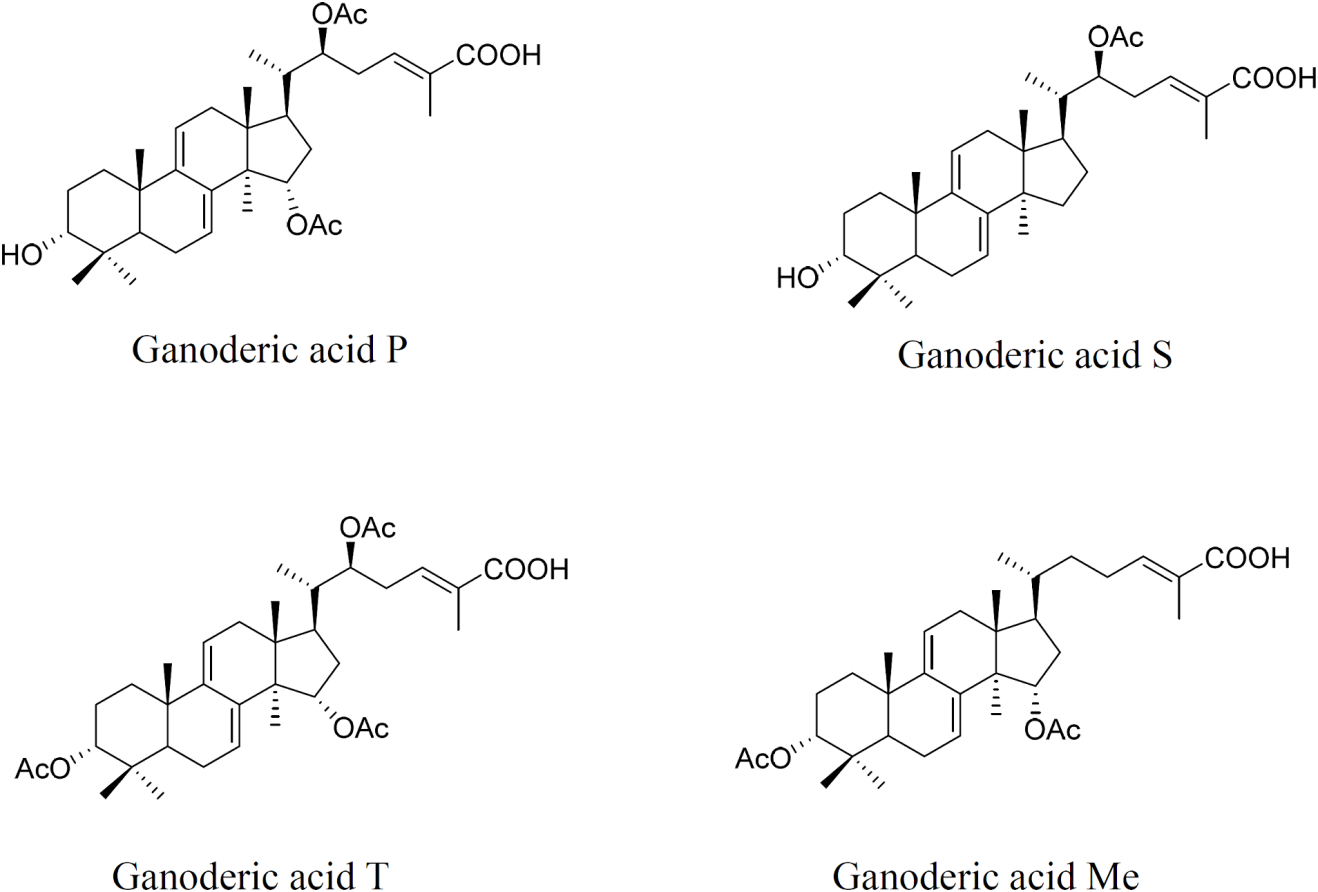
Chemical structures of individual ganoderic acid-P, ganoderic acid-S, ganoderic acid-T, and ganoderic acid-Me.

Samples were collected at the mycelial, the primordial, and fruiting body stages. Individual GAs, lanosterol, and squalene were extracted and determined using a 1,260 Infinity II HPLC with a 5 μm Zorbax Extend-C18 column (4.6 × 250 mm) and UV/DAD as previously described (Xu et al., 2019; Yue et al., 2023). The individual GAs (Figure 1), lanosterol, and squalene contents were quantified by referencing calibration curves created using different standard solutions and according to dry weight.

### 2.7 Statistical analysis

All the data are presented as the average of three biological replicates, and the error bars indicate the standard deviation of three replicates. The data were analyzed by one-way analysis (ANOVA) of variance using SPSS (version 24.0, Chicago, IL, United States). A *p*-value <0.05 was considered to indicate significance.

## 3 Results

### 3.1 Isolation and identification of the monokaryotic strains G. 260124 and G. 260125

**Figure 2:**
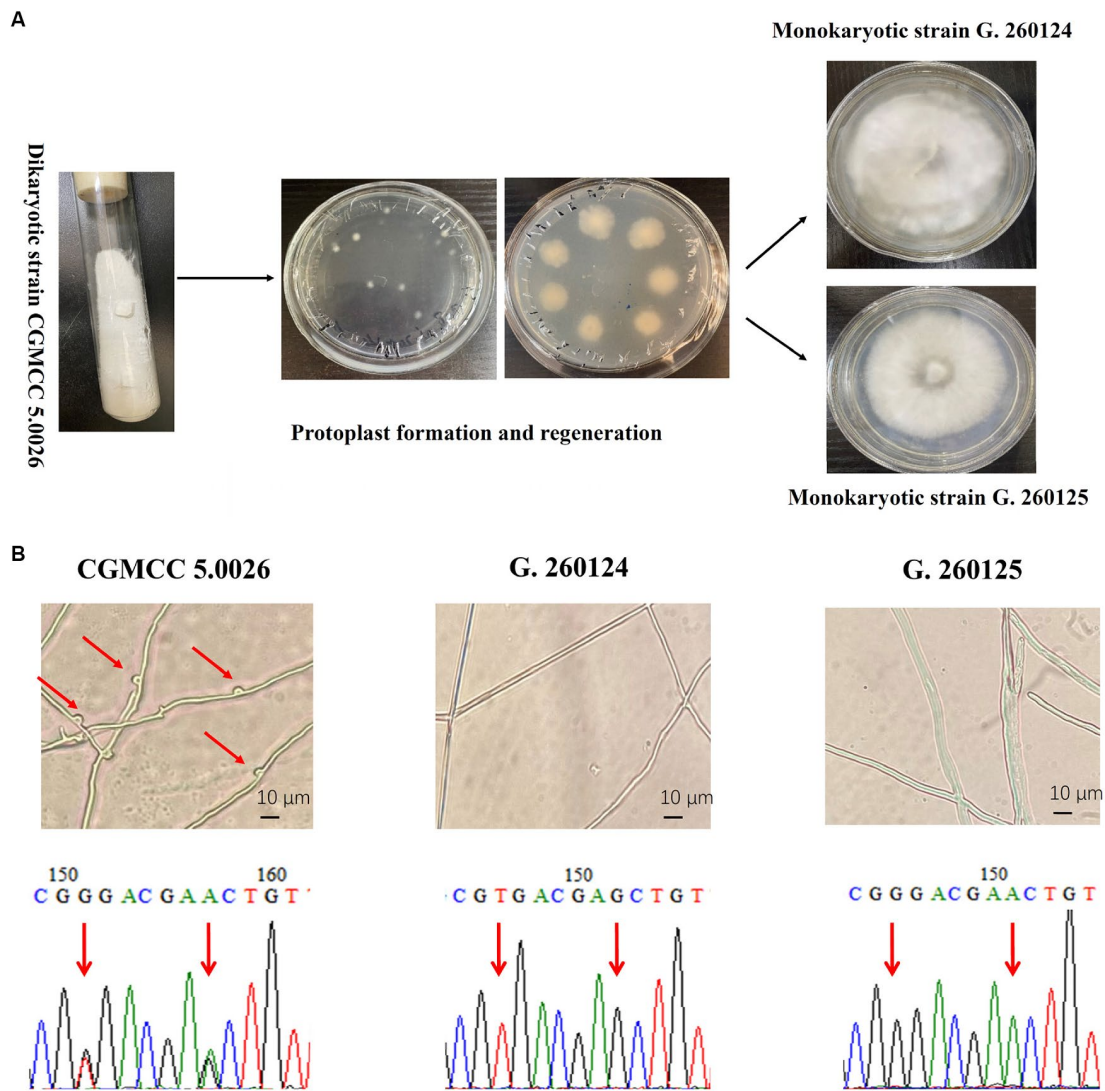
Isolation and identification of monokaryotic *G. lucidum* strains. **(A)** Isolation of the monokaryotic strain G. 260124 and G. 260125 from the dikaryotic CGMCC 5.0026 strain by protoplast formation and regeneration. **(B)** Identification of the monokaryotic strains G. 260124 and G. 260125 by morphological observation and SNP-PCR. Clamp connections and two SNPs are shown.

The monokaryotic strains G. 260124 and G. 260125 were prepared from the dikaryotic strain CGMCC 5.0026 by protoplast regeneration as described by Sun et al. (2021) (Figure 2A). After isolating protoplast-regenerated colonies from the dikaryotic CGMCC 5.0026 strain, mycelia from these colonies were analyzed via clamp connections and SNP-PCR. Each regenerated colony was either a dikaryon or a monokaryon. The presence of clamp connections was observed in the CGMCC 5.0026 strain. However, no clamp connections were found for the G. 260124 or G. 260125 strains. Additionally, SNP-PCR analysis confirmed that the G. 260124 and G. 260125 strains were monokaryotic *G. lucidum* strains (Figure 2B). Two SNPs in the dikaryotic strain CGMCC 5.0026 were confirmed by sequencing the PCR products, but no SNPs were found when the sequenced PCR products were amplified from the G. 260124 and G. 260125 strains. These results showed that two monokaryons were successfully isolated from the dikaryotic *G. lucidum* strain CGMCC 5.0026.

### 3.2 Generation of the transgenic *Ganoderma lucidum* G. 260125-sqs strain and G. 260124-vgb expressing *vgb*

**Figure 3:**
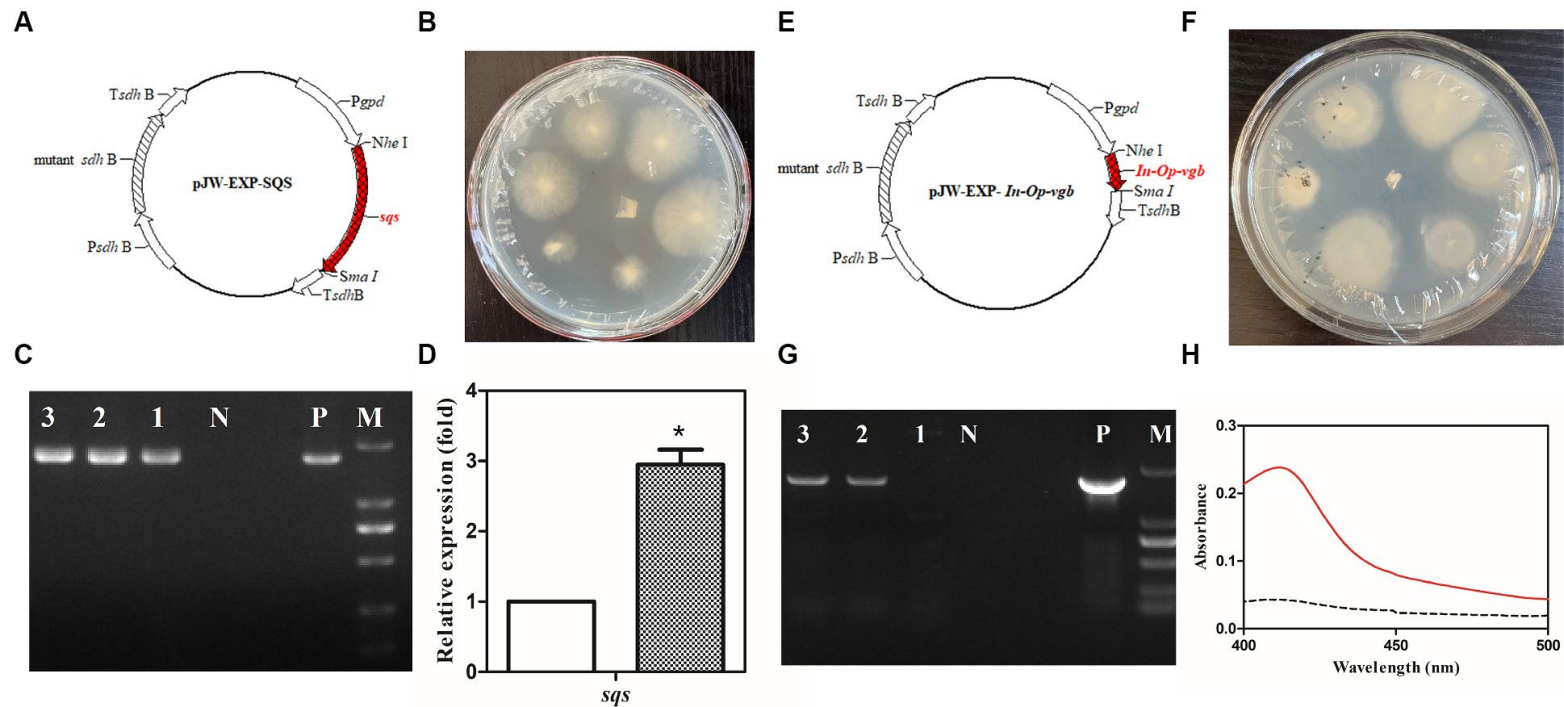
Generation and verification of the transgenic *G. lucidum* strain G. 260125-sqs and G. 260124-vgb. Structures of the plasmids pJW-EXP-SQS **(A)** and pJW-EXP-In-Op-vgb **(E)**. Selection of the transgenic strains G. 260124-vgb **(B)** and G. 260125-sqs **(F)** on a CYM selection plate. Identification and characterization of the transgenic strains G. G. 260124-vgb **(C)** and G. 260125-sqs **(G)** by genome PCR. **(D)** Transcriptional levels of the *sqs* in the CGMCC 5.0026 (blank) and the G. 260125-sqs (gray) strains. **(H)** CO-difference spectra analysis of the CGMCC 5.0026 (dotted line) and G. 260124-vgb (solid line) strains. Lane P, the plasmid positive control; Lane N, negative control; Lanes 1, 2, 3, the transgenic strains; Lane M, DNA marker DL2000.

Protoplasts of the *G. lucidum* G. 260125 and G. 260124 strains were genetically transformed with the plasmids pJW-EXP-SQS (Figure 3A) and pJW-EXP-*In-Op-vgfb* (Figure 3E) as previously described (Yu et al., 2014; Xu et al., 2023), respectively. The transformation efficiency was 15–20 transformants per μg DNA per 10^7^ protoplasts. Candidate G. 260125-sqs (Figure 3B) and G. 260124-vgb transformants (Figure 3F) were selected on CYM plates supplemented with 2 mg/L carboxin antibiotics after five rounds of growth on fresh CYM plates. The integration of the target gene into the transformants was verified by genome PCR. A clear band for the fused *gpd* promoter and the target gene (−1.9 kb for *sqs* or −1.7 kb for *vgb*) was present in the transformants G. 260125-sqs and G. 260124-sqs, whereas no corresponding band was observed in the control strain (Figures 3C,G). qRT-PCR analysis revealed that *sqs* was overexpressed in the transformant G. 260125-sqs at an approximately 2.95-fold higher level than that in the G. 260125 strain (Figure 3D). The activity of the *Vitreoscilla* hemoglobin in the transformant G. 260124-vgb was analyzed via the carbon monoxide difference spectrum method. A maximum characteristic absorbance at 418 nm was detected for the crude extract of the transformant G. 260124-vgb, but no such absorbance was observed for the crude extract of the G. 260124 strain (Figure 3H). Our results illustrate that *Vitreoscilla* hemoglobin is biologically active in the transgenic strain G. 260124-vgb.

### 3.3 Dikaryotic strains produced by mono–mono crossing of the G. 260125-sqs and G. 260124-vgb strains

**Figure 4:**
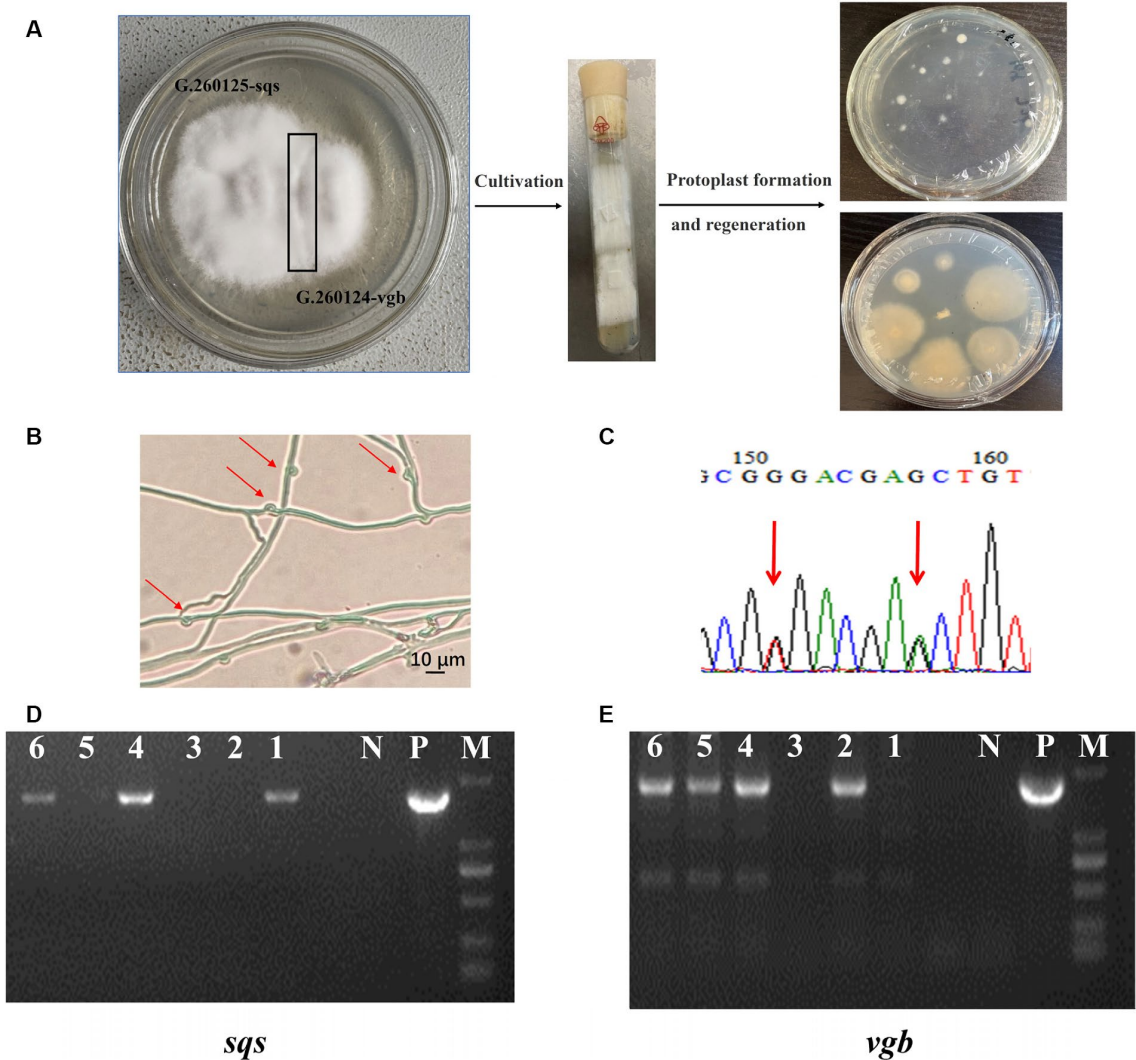
Generation and verification of the dikaryotic *G. lucidum* strain sqs-vgb by mating mycelia of the G. 260125-sqs and G. 260124-vgb strains. **(A)** Cross mating of two monokaryon G. 260125-sqs and G. 260124-vgb, and regeneration of protoplasts from the mycelia from the contact area. Identification of the dikaryotic strain sqs-vgb by morphological observation **(B)** and SNP-PCR **(C)**. The integration of *sqs* **(D)** and *vgb* **(E)** in the genome of the selected sqs-vgb strain was confirmed via PCR. Clamp connections and two SNPs are shown. Lane P, the plasmid positive control; Lane N, negative control; Lanes 1, 2, 3, 4, 5, and 6, the selected sqs-vgb strains; Lane M, DNA marker DL2000.

Mono–mono crossing was conducted by mating the G. 260125-sqs and G. 260124-vgb strains (Figure 4A). Mycelia from the contact area between the monokaryotic mycelia of the G. 260125-sqs and G. 260124-vgb strains were picked and cultured on PDA slants. Subsequently, protoplasts of mycelia from the PDA slants were regenerated on CYM plates in the dark. The integration of *sqs* and *vgb* into the genome of the protoplast-regenerated colonies was determined by genome PCR. The expected products of 1.9 kb and 1.7 kb, were present in colonies No. 4 and 6, respectively (Figures 4D,E). Then, protoplast-regenerated colonies No. 4 and 6 were selected to observe clamp connections and perform SNP-PCR analysis. The selected strains developed clamp connections (Figure 4B). In addition, two overlapping peaks were found in the SNP sites in the mitochondrial intermediate peptidase gene of the selected colonies (Figure 4C). These results indicate that the mated *G. lucidum* sqs-vgb strain is a dikaryon overexpressing both *sqs* and *vgb*.

### 3.4 Increased contents of individual GAs in the fruiting body of the *Ganoderma lucidum* sqs-vgb strain

**Figure 5:**
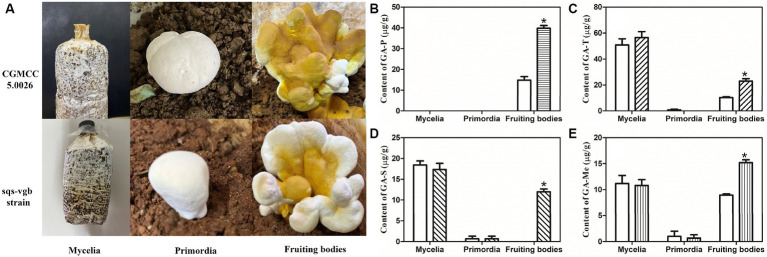
Growth of the CGMCC 5.0026 and sqs-vgb strains **(A)** and contents of individual GA-P **(B)**, GA-T **(C)**, GA-S **(D)**, and GA-Me **(E)** at three different developmental stages of the CGMCC 5.0026 (blank) and sqs-vgb strains (filled). ^*^A significantly different from the CGMCC 5.00296 strain.

The cultivation status of the *G. lucidum* sqs-vgb strain was compared with that of the control strain CGMCC 5.0026 (Figure 5A). The mycelia of the mated strain sqs-vgb were similar to those of the control strain. The times for spawn running (15 days), primordium formation (25 days), and fruiting body formation (65–75 days) in the sqs-vgb strain were comparable to those in the control strain. No significant morphological differences were observed between the fruiting bodies of the sqs-vgb and control strains. This might be attributable to the same nuclear background between the two strains.

The accumulation of individual GAs at three different developmental stages was then investigated in the *G. lucidum* sqs-vgb and control strains. The sqs-vgb strain exhibited a significant increase in the content of individual GAs in the fruiting body (Figures 5B-E). The maximum content of GA-P measured in the sqs-vgb strain was 39.8 μg/g DW, an increase of approximately 2.69-fold compared with that in the control strain. However, no GA-P was detected at the mycelia or primordia stage in either strain. The contents of GA-T and GA-Me at the mycelia and fruiting body stages were much greater than those at the primordia stage. The maximum contents of GA-T and GA-Me in the sqs-vgb strain at the fruiting body stage were 23.1 μg/g DW and 15.3 μg/g DW, respectively, 2.23- and 1.75-fold greater than those in the control strain. GA-S was detected only at the mycelia and primordia stages in the control strain; however, the sqs-vgb strain accumulated GA-S at all three developmental stages. The maximum GA-S content in the fruiting body of the sqs-vgb strain was 11.9 μg/g DW, while no GA-S was detected in the fruiting body of the control strain.

### 3.5 Accumulation of intermediates and expression of GA biosynthetic genes in the fruiting body of the *Ganoderma lucidum* sqs-vgb strain

**Figure 6:**
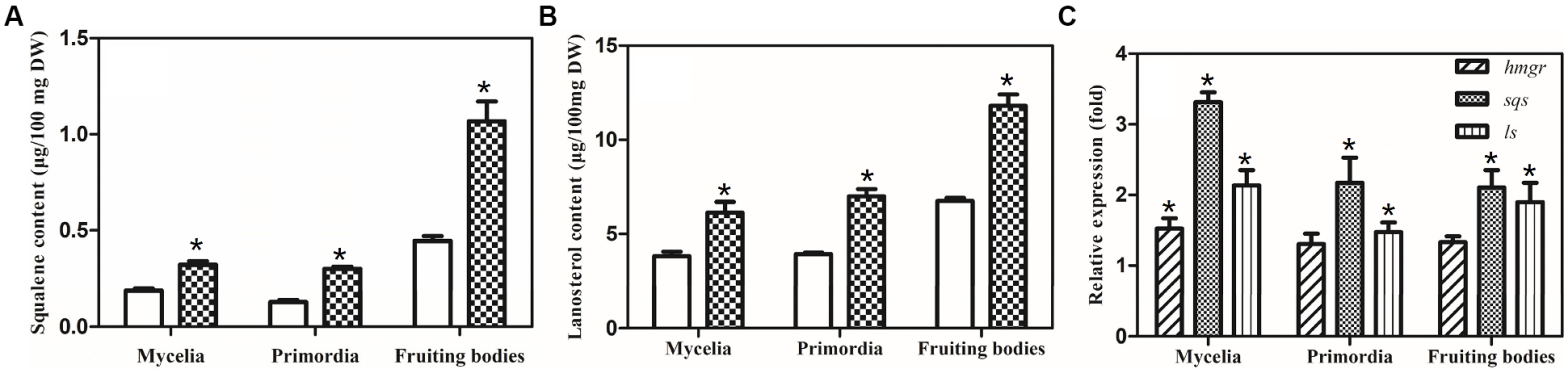
Intermediate accumulations of and gene expression levels of GA biosynthetic genes at three different developmental stages of the CGMCC 5.0026 and sqs-vgb strains. Accumulation of squalene **(A)** and lanosterol **(B)** in the CGMCC 5.0026 (blank) and sqs-vbg (filled) strains. Transcriptional levels of *hmgr*, *sqs* and *ls* at three developmental stages of the CGMCC 5.0026 and sqs-vgb **(C)**. The expression of genes in the CGMCC 5.0026 strain was defined as 1.0, and the expression levels in the sqs-vgb strain are shown as fold changes compared to the reference. ^*^A significantly different from the CGMCC 5.00296 strain.

Squalene and lanosterol are important intermediates in the GA biosynthetic pathway; thus, the accumulation of squalene and lanosterol was analyzed in the control and sqs-vgb. The accumulations of squalene and lanosterol increased during development from the mycelia to the fruiting bodies in both the control and sqs-vgb strains (Figures 6A,B). The maximum levels of squalene and lanosterol in the fruiting body of the *G. lucidum* sqs-vgb strain were 1.06 μg/100 mg DW and 11.8 μg/100 mg DW, 2.35- and 1.75-fold greater than those in the control strain, respectively. At three different stages of fruiting body development, more squalene and lanosterol accumulated in the sqs-vgb strain than in the control strain. The expression levels of *hmgr*, *sqs*, and *ls* were also determined through qRT-PCR at three stages of fruiting body development in both strains. The expression levels of *sqs* and *ls* were higher in the sqs-vgb strain than in the control strain (Figure 6C). The maximum expression levels of *sqs* and *ls* in the sqs-vgb strain were 3.23- and 2.13-fold greater than those in the control strain, respectively.

## 4 Discussion

In this study, the monokaryotic *G. lucidum* G. 260124 and G. 260125 strains were obtained from the dikaryotic 5.0026 strain through protoplast technology. These mononucleate protoplasts of *G. lucidum* can be isolated from monokaryotized cells on the top of the hyphae of dikaryotic mycelia (Maeta et al., 2008). The protoplasted monokaryotic *G. lucidum* strain provides unique parents for mono–mono crossing. This method may be easier and faster than the use of the sporulated monokaryotic isolates. In particular, this method is useful for crossbreeding these mushrooms because the low germination rate makes it challenging to obtain monokaryotic isolates from basidiospores. A previous study demonstrated that the basidiospores of *Ganoderma* are difficult to germinate under artificial culture conditions (Liu et al., 2017). Our results will be useful for *Ganoderma* breeding and strain improvement.

Crossbreeding is historically considered the most efficient way to develop new strains of edible mushrooms (Kim et al., 2013; Sonnenberg et al., 2017). When monokaryotic mycelia with different *MAT* genotypes are mated, the characteristics of the hybrids will inherit the preferred characteristics of the parent strains (Avin et al., 2016). Therefore, the benefits of crossbreeding depend on the specific parent strains. Our previous works demonstrated that the expression of either *vgb* or *sqs* led to an increased accumulation of individual GAs, including GA-S, GA-T, and GA-Me, in submerged cultures of *G. lucidum*. Consequently, we explored the effects of manipulating both the *vgb* and *sqs* on the accumulation of these individual GAs in the fruiting body of *G. lucidum*. In the present work, *sqs* was introduced into the parent strain *G. lucidum* G. 260125 strain, and *vgb* was heterologously expressed in the other parent strain, G. 260124. In the area where the two monokaryotic mycelia contact, there may be three different types of mycelia: the G. 260124-vgb, the G. 260125-sqs, and the mated *G. lucidum* sqs-vgb. In order to isolate single colonies of the dikaryotic strain sqs-vgb, protoplasts derived from the mycelia in the contact area were regenerated on CYM plates. The mycelia derived from these regenerated protoplasts were screened through the observation of clamp connections and an SNP-PCR analysis. Then, a new *G. lucidum* sqs-vgb strain was generated by mono–mono crossing the G. 260124-vgb and G. 260125-sqs strain. An increase in the contents of individual GAs in the sqs-vgb strain was achieved by mono-crossing the G. 260124-vgb and G. 260125-sqs monokaryons. To our knowledge, this is the first study on the breeding of *G. lucidum* by integrating genetic modification and mono–mono crossing. Our results suggest that integrating genetic engineering and mono–mono crossing is an effective strategy for enhancing the individual GA content in the fruiting body of *G. lucidum*. Two monokaryotic compatible hyphae can fuse and give rise to a dikaryotic mycelium in which the two parental nuclei are independent throughout vegetative growth, and the mycelia will fruit under the appropriate conditions. Additionally, the combination of genetic engineering and mono–mono crossing allows the transformation of multiple genes into *G. lucidum* with a limited number of selection marker genes. This strategy will be useful for manipulating of the biosynthetic pathway of secondary metabolites in *Ganoderma*.

The increased contents of individual GAs in the mated sqs-vgb strain may be related to the fact that the beneficial traits of both monokaryons were transferred to the dikaryotic strain. Our results show that *sqs* overexpression and heterologous expression of *vgb* increase the supply of squalene and lanosterol, potentially increasing the conversion of intermediates into individual GAs. Previous studies also showed that heterologous expression of *vgb* or *sqs* overexpression upregulates the transcription levels of *sqs* and *ls* in submerged cultures of *G. lucidum* (Zhou et al., 2014; Li et al., 2016b). The increased contents of individual GAs in the mated sqs-vgb strain may be attributable to the improved precursor supply and the upregulated expression of *sqs* and *ls*. These results also align with those of previous studies (Zhang et al., 2017b; Ye et al., 2018; Xu et al., 2019), where increased production of GAs coincided with enhanced accumulation of intermediates and expression of GA biosynthetic genes. In addition, our results also showed that the contents of individual GAs changed during fruiting body development in *G. lucidum*, suggesting that the biosynthesis of individual GAs may be correlated with mushroom development. Previous studies have shown that triterpene components differ among the different developmental stages of *G. lucidum* (Hirotani and Furuya, 1990; Nakagawa et al., 2018).

## 5 Conclusion

In the present study, a high-GA-producing *G. lucidum* strain was obtained by cross-mating monokaryotic *G. lucidum* G. 260124 overexpressing *vgb* with G. 260125 expressing *sqs*. The contents of GA-T, -S, -Me, and -P were significantly greater in the fruiting bodies of the mated *G. lucidum* sqs-vgb strain than in those of the control CGMCC 5.0026 strain. The enhanced intermediate supply and expression levels of GA biosynthetic genes may have increased the contents of individual GAs in the mated strain. We demonstrated that an integrated approach that combines genetic engineering and mono–mono crossing can effectively improve the accumulation of individual GAs in the fruiting body of *G. lucidum*. These findings will be useful for breeding *Ganoderma* and hyperproducing of individual GAs in the fruiting body of *G. lucidum*.

## Data availability statement

The original contributions presented in the study are included in the article/supplementary material, further inquiries can be directed to the corresponding authors.

## Author contributions

D-XZ: Investigation, Methodology, Validation, Writing – original draft. X-MK: Investigation, Methodology, Validation, Writing – original draft. X-MH: Investigation, Methodology, Validation, Writing – review & editing. NL: Data curation, Methodology, Validation, Writing – review & editing. NF: Conceptualization, Methodology, Writing – review & editing. J-WX: Conceptualization, Data curation, Funding acquisition, Writing – original draft, Writing – review & editing.
